# Safety and Outcomes of Concomitant Left Atrial Appendage Occlusion and Atrial Fibrillation Ablation

**DOI:** 10.1016/j.jacadv.2026.102870

**Published:** 2026-06-11

**Authors:** Khalid Sawalha, Saeed Abughazaleh, Ahmad Al Nawaiseh, Kyle Gobeil, Marshal Fox, E. Kevin Heist, Fadi Chalhoub

**Affiliations:** a Department of Cardiovascular Medicine, Baystate Medical Center and Division of Cardiovascular Medicine, University of Massachusetts-Baystate, Springfield, Massachusetts, USA; b Division of Cardiovascular Disease, University of Connecticut School of Medicine/ Hartford Hospital, Hartford, Connecticut, USA; c Demoulas Center for Cardiac Arrhythmias Massachusetts General Hospital Harvard Medical School, Boston, Massachusetts, USA

**Keywords:** AF ablation, concomitant procedure, LAAO, safety

## Abstract

**Background:**

Performing atrial fibrillation (AF) catheter ablation and percutaneous left atrial appendage occlusion (LAAO) during a single procedure may improve efficiency and reduce cumulative procedural risk. However, concerns remain regarding potential increases in periprocedural and longer-term complications, including stroke, pericardial effusion, device leak, and device-related thrombus. Evidence evaluating the safety of this concomitant strategy remains limited.

**Objectives:**

The objectives of the study was to compare the safety and short- and long-term outcomes of concomitant AF ablation plus LAAO vs LAAO alone.

**Methods:**

We conducted a retrospective cohort study using the TriNetX U.S. Collaborative Network, identifying adults (≥18 years) with AF who underwent percutaneous LAAO between January 2015 and February 2026. Patients undergoing AF ablation on the same day as LAAO were compared with those undergoing LAAO alone. After 1:1 propensity score matching for demographics, comorbidities, cardiovascular medications, and laboratory values, 1,899 patients were included in each group. Outcomes including all-cause mortality, stroke, device leak, device-related thrombus, pericardial effusion, and pericardiocentesis were assessed at 7 days, 90 days, and 1 year using cumulative incidence and Cox proportional hazards models.

**Results:**

Baseline characteristics were well balanced after matching. Concomitant AF ablation was not associated with increased adverse events. Mortality was similar at 90 days (0.6% vs 0.6%; HR: 1.00; 95% CI: 0.43-2.32) and 1 year (1.4% vs 1.5%; HR: 0.97; 95% CI: 0.58-1.63). At 90 days, rates of stroke, device leak, and device-related thrombus were comparable between groups, with no differences observed at 1 year.

**Conclusions:**

In this large, propensity-matched analysis, concomitant AF ablation at the time of LAAO was not associated with increased early or 1-year complications, supporting the safety of a combined approach in appropriately selected patients.

## Graphical Abstract

**Figure:**
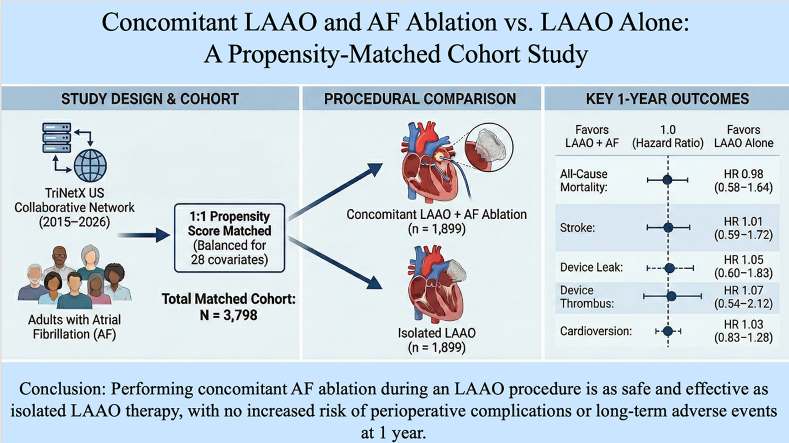

Atrial fibrillation (AF) is the most common sustained cardiac arrhythmia and is associated with an increased risk of ischemic stroke, heart failure, impaired quality of life, and mortality.^1^ Contemporary guidelines advocate for an integrated approach to AF management that incorporates rate and rhythm control strategies alongside stroke prevention.^2^ Although catheter ablation is an effective rhythm control therapy, current guidelines recommend indefinite oral anticoagulation (OAC) in patients with moderate to high stroke risk, even after apparently successful ablation, due to the potential for asymptomatic AF recurrence.^3^ Despite these recommendations, up to one-quarter of patients discontinue OAC within 1 year, most commonly due to bleeding risk, intolerance, or cost.^4^^,^^5^

Percutaneous left atrial appendage occlusion (LAAO) has emerged as an effective nonpharmacologic alternative for stroke prevention in patients with nonvalvular AF who have contraindications to long-term OAC or are at high bleeding risk.^6^ Large registries and meta-analyses have demonstrated high procedural success rates and low rates of major complications, despite the elevated baseline risk of both stroke and bleeding in this population.^7^^,^^8^

Both AF catheter ablation and LAAO are invasive procedures requiring transseptal access to the left atrium and are therefore associated with procedural risks, including vascular complications and pericardial effusion. Traditionally, when both procedures are indicated, they are performed in a staged manner, often due to the long procedure time of AF ablation especially with radiofrequency ablation. However, this approach necessitates repeated venous and left atrial access, potentially increasing cumulative procedural risk and health care utilization. In addition, a period of OAC is generally required following AF ablation, which may expose high-risk patients to bleeding complications while awaiting LAAO.

With advancements in ablation technologies, imaging, and procedural workflows leading to shorter procedure times, the performance of concomitant AF ablation and LAAO during a single session has become increasingly feasible. Prior studies suggest that concomitant AF ablation and Watchman device implantation can be performed safely, with procedural success rates ranging from 87% to 99% and complication rates comparable to those of standalone procedures.^9^ Despite growing interest in the “one-stop” strategy of concomitant AF ablation and LAAO, existing evidence is largely limited to small or modest cohorts, often without appropriate comparator groups and with short follow-up. Consequently, the incremental procedural risk compared with LAAO alone remains incompletely defined, and contemporary real-world data are lacking. To address these gaps, we conducted a large, real-world, propensity score–matched analysis using a national electronic health record network to compare outcomes of concomitant AF ablation plus LAAO vs LAAO alone across multiple clinically relevant time horizons.

## Methods

We conducted a retrospective, observational cohort study using the TriNetX U.S. Collaborative Network, a federated research platform that aggregates deidentified electronic health record data from participating health care organizations across the United States. The database includes demographics, diagnoses, procedures, medications, laboratory values, and outcomes. As all data are deidentified, this study was exempt from institutional review board approval and informed consent requirements.

### Study population and cohort definitions

Adult patients (≥18 years) with a diagnosis of AF who underwent percutaneous LAAO on or after January 1, 2015, to February 1, 2026, were eligible for inclusion. Two cohorts were defined based on procedural timing: patients who underwent catheter ablation for AF on the same day as LAAO constituted the concomitant LAAO plus AF ablation cohort, whereas patients who underwent LAAO without concomitant AF ablation comprised the LAAO-alone cohort. The index date was defined as the date of the LAAO procedure for both cohorts. Patients whose qualifying procedures occurred 20 years or more before the index date were excluded in accordance with TriNetX analytic constraints.

### Outcomes

Clinical outcomes were assessed at 3 prespecified time horizons: periprocedural (7 days), short-term (90 days), and long-term (1 year) following the index date. Outcomes of interest included all-cause mortality, ischemic stroke, device leak, device-related thrombus, pericardial effusion, cardiac tamponade, and pericardiocentesis. For each outcome, patients with a documented history of that outcome before the index date were excluded from the corresponding analysis to ensure evaluation of incident events.

### Propensity score matching

To account for baseline differences between cohorts, 1:1 propensity score matching was performed using a nearest-neighbor algorithm with a caliper of 0.1 pooled SDs. Matching variables included demographics, cardiovascular and noncardiovascular comorbidities, baseline cardiovascular medications, and relevant laboratory values (Table 1). Balance between cohorts was assessed using standardized mean differences, with values <0.1 indicating adequate balance. After matching, 1,899 patients remained in each cohort.

**Table 1:** Baseline Characteristics After Propensity Score Matching∗

	Concomitant LAAO + AF Ablation (n = 1,899)	LAAO Alone (n = 1,899)	SMD
Age, years	73.8 ± 7.6	73.9 ± 7.6	0.002
Female	773 (40.7%)	775 (40.8%)	0.002
White race	1,647 (86.7%)	1,648 (86.8%)	0.002
Black or African American	127 (6.7%)	126 (6.6%)	0.002
Hispanic or Latino	31 (1.6%)	31 (1.6%)	<0.001
Asian	53 (2.8%)	53 (2.8%)	<0.001
Heart failure	701 (36.9%)	701 (36.9%)	<0.001
Ischemic heart disease	872 (45.9%)	873 (46.0%)	0.001
Hypertension	1,350 (71.1%)	1,350 (71.1%)	<0.001
Chronic kidney disease	440 (23.2%)	439 (23.1%)	0.001
Diabetes mellitus	513 (27.0%)	514 (27.1%)	0.001
Peripheral vascular disease	137 (7.2%)	137 (7.2%)	<0.001
Beta-blocker use	1,329 (70.0%)	1,328 (69.9%)	0.001
ACE inhibitor use	300 (15.8%)	298 (15.7%)	0.003
ARB use	641 (33.8%)	643 (33.9%)	0.002
Apixaban	1,268 (66.8%)	1,270 (66.9%)	0.002
Warfarin	95 (5.0%)	95 (5.0%)	<0.001
Hemoglobin, g/dL	13.2 ± 2.0	13.2 ± 2.0	0.018
Creatinine, mg/dL	1.2 ± 1.0	1.2 ± 1.0	0.002
LVEF, %	55.3 ± 10.8	55.5 ± 10.7	0.013

ACE = angiotensin-converting enzyme; AF = atrial fibrillation; ARB = angiotensin receptor blocker; LAAO = left atrial appendage occlusion; LVEF = left ventricular ejection fraction; SMD = standardized mean difference.

∗Propensity score matching achieved excellent covariate balance, with all standardized mean differences <0.1, indicating minimal residual imbalance between cohorts.

Data are presented as mean ± SD or n (%)

### Statistical analysis

Categorical variables were summarized as counts and percentages, and continuous variables as means with SD. Mortality rates were estimated for each cohort using Kaplan-Meier methods with censoring at last follow-up, whereas nonfatal outcomes were evaluated using cumulative incidence estimates considering death as a competing risk. Cause-specific cox proportional hazards models were used to estimate HRs and 95% CIs. Time-to-event analyses were performed using Kaplan-Meier estimates. For the Cox proportional hazards model, we assessed whether the proportional hazards assumption was held by applying a chi-square test based on Schoenfeld residuals. These statistical outputs, including the HR and its CI, were generated using TriNetX proprietary cloud-based analytic algorithms tool (R version 4.0.2; R project for statistical computing), and a 2-sided *P* value <0.05 was considered statistically significant.

## Results

A total of 1,899 patients undergoing concomitant LAAO and AF ablation were matched 1:1 to patients undergoing LAAO alone using propensity score matching. Baseline demographic characteristics, comorbidities, medication use, and laboratory values were well balanced between cohorts after matching, with all standardized mean differences <0.1 (Table 1, Cental Illustration).

### Periprocedural outcomes (7 days)

Periprocedural clinical events were infrequent in both groups (Table 2). All-cause mortality was <0.1% in each cohort. Stroke occurred in 0.6% of patients in both groups (HR: 1.09; 95% CI: 0.48-2.47). Pericardial effusion: defined as effusions of any size identified either intraprocedurally or during postprocedural follow-up, was observed in 6.8% of patients in the concomitant group and 6.7% in the LAAO-alone group (HR: 1.02; 95% CI: 0.80-1.30). No tamponade or pericardiocentesis occurred within 7 days.

**Table 2:** Periprocedural (7-Day) Outcomes After AF Ablation With and Without LAAO

	LAAO + AF Ablation (n = 1,899)	LAAO Alone (n = 1,899)	HR (95% CI)
Periprocedural outcomes, 7 d %			
All-cause mortality	<0.1	<0.1	Not estimable^a^
Stroke	0.6	0.6	1.09 (0.48-2.47)
Pericardial effusion^b^	6.8	6.7	1.02 (0.80-1.30)
All-cause hospitalization	12.6	12.6	1.00 (0.84-1.20)
Short-term outcomes, 90 d%			
All-cause mortality	0.6	0.6	1.00 (0.43-2.32)
Stroke	0.9	0.9	1.00 (0.48-2.08)
Pericardial effusion	8.2	8.2	1.00 (0.80-1.25)
Tamponade	0.4	0.4	1.00 (0.42-2.37)
Device leak	0.9	0.8	1.06 (0.53-2.10)
Device-related thrombus	0.6	0.5	1.10 (0.47-2.57)
Cardioversion	7.3	7.2	1.02 (0.81-1.28)
All-cause hospitalization	13.6	13.6	1.00 (0.85-1.18)
Long-term outcomes, 1 y%			
All-cause mortality	1.4	1.5	0.97 (0.58-1.63)
Stroke	1.4	1.4	1.00 (0.58-1.72)
Device leak	1.3	1.2	1.04 (0.59-1.83)
Device-related thrombus	0.9	0.8	1.06 (0.54-2.09)
Cardioversion	8.6	8.5	1.02 (0.83-1.26)

Values are presented as estimated event rates (%). Mortality rates were estimated using Kaplan-Meier methods with censoring at last follow-up. Nonmortality outcomes were estimated using cumulative incidence methods accounting for death as a competing risk. HRs are presented with 95% CIs.

Abbreviations as in Table 1.

a HR not estimable because of very low event counts.

b Pericardial effusion: Includes pericardial effusions of any size identified either intraprocedurally or during postprocedural follow-up.

### Short-term outcomes (90 days)

At 90 days, all-cause mortality remained low and identical between cohorts (0.6% vs 0.6%; HR: 1.00; 95% CI: 0.43-2.32). Stroke occurred in 0.9% of patients in each group (HR: 1.00; 95% CI: 0.48-2.08). Rates of pericardial effusion were similar (8.2% vs 8.2%; HR: 1.00; 95% CI: 0.80-1.25), as were tamponade (0.4% vs 0.4%; HR: 1.00; 95% CI: 0.42-2.37), device leak (0.9% vs 0.8%; HR: 1.06; 95% CI: 0.53-2.10), and device-related thrombus (0.6% vs 0.5%; HR: 1.10; 95% CI: 0.47-2.57). All-cause hospitalization rates were also comparable (13.6% vs 13.6%; HR: 1.00; 95% CI: 0.85-1.18).

### Longer-term outcomes

During longer-term follow-up, outcomes remained similar between cohorts. All-cause mortality occurred in 1.4% and 1.5% of patients, respectively (HR: 0.97; 95% CI: 0.58-1.63). Stroke occurred in 1.4% of patients in both groups (HR: 1.00; 95% CI: 0.58-1.72). Device leak (1.3% vs 1.2%; HR: 1.04; 95% CI: 0.59-1.83) and device-related thrombus (0.9% vs 0.8%; HR: 1.06; 95% CI: 0.54-2.09) did not differ between groups. Overall, across periprocedural, short-term, and 1-year follow-up, concomitant AF ablation performed at the time of LAAO was not associated with an increased risk of mortality, thromboembolic events, or device- or pericardial-related complications compared with LAAO alone.

## Discussion

Our analysis showed that performing AF ablation concomitantly with LAAO was not associated with an increased risk of periprocedural, short-term, or 1-year adverse clinical outcomes compared with LAAO alone. Across all evaluated time horizons up to 1 year postprocedure, rates of mortality, ischemic stroke, device leak, device-related thrombus, and pericardial complications were low and comparable between groups. These findings support the procedural safety of a combined strategy in appropriately selected population.

Our findings are consistent with and extend prior reports demonstrating high procedural success and favorable safety profiles for concomitant AF ablation and LAAO. Registry data from 11 centers involving 142 patients reported successful LAAO in over 99% of cases when performed during the same session as AF ablation.^10^ Similarly, a large Chinese cohort reported satisfactory device sealing in all patients undergoing combined procedures.^11^ Moreover, a meta-analysis showed that combined RF ablation and LAAO achieves procedural success rates approaching 98% to 100%, with stroke/TIA rates of approximately 1% and device-related thrombus rates of 0% to 1% during long-term follow-up.^12^

### Periprocedural safety

When interpreting the safety of a combined approach, it is important to consider the established risk profiles of AF ablation and LAAO as individual procedures. Meta-analyses of LAAO devices, including Watchman and Amplatzer/Amulet, report periprocedural pericardial effusion rates ranging from 2% to 4%, tamponade rates of approximately 1% to 2%, and device embolization rates below 2%, with no statistically significant differences between devices. Similarly, large U.S. registry data evaluating AF catheter ablation report overall complication rates of approximately 5%, with life-threatening events occurring in fewer than 1% of patients.^13^ In contrast, a meta-analysis of 18 studies including 1,385 patients undergoing combined AF ablation and LAAO demonstrated pooled incidences of pericardial effusion, bleeding events, and residual flow of 0.5%, 1.4%, and 7.2%, respectively, which are comparable to or lower than those reported for the individual procedures.^14^

In our analysis, clinically significant postprocedural complications were rare. Tamponade and pericardiocentesis occurred in fewer than 1% of patients and did not differ between groups. Although the observed rate of pericardial effusion was relatively higher, this likely reflects the inherent limitations of large administrative databases. The data set does not differentiate whether effusions were present before the procedure or detected incidentally postprocedure. In addition, it does not specify whether these represented clinically significant effusions or only small or trace effusions. Most important, no increase in effusions requiring pericardiocentesis was observed during the procedure day or within the 7-day periprocedural period, suggesting that clinically meaningful pericardial complications were uncommon. Collectively, these findings indicate that a single-session approach does not appear to confer additive procedural risk.

### Thromboembolic, device-related, and long-term outcomes

A major concern surrounding concomitant AF ablation and LAAO is the potential for increased thromboembolic risk especially with the increased left atrial dwell time and postablation atrial stunning. In our analysis, rates of ischemic stroke and device-related thrombus were low and did not differ significantly between the concomitant and LAAO alone cohorts at either 90 days or 1 year.

Although calculation of an individualized CHA_2_DS_2_-VASc score is challenging in large real-world databases, we performed rigorous propensity score matching incorporating the major clinical components of the score and thromboembolic risks. The balanced distribution of these variables between groups supports the validity of our comparison and strengthens the inference that thromboembolic risk was similar across both approaches.

These findings are consistent with contemporary randomized evidence. The OPTION trial demonstrated that LAAO with the Watchman device provides stroke prevention comparable to OAC while significantly reducing nonprocedural bleeding, irrespective of whether LAAO was performed concomitantly with AF ablation or staged 90 to 180 days later.^15^ Both procedural timing strategies achieved similar efficacy and safety outcomes. In our cohort, device-related thrombosis and peridevice leak rates were infrequent (<1%) and comparable between groups, further supporting that a concomitant approach does not adversely affect device performance or long-term thromboembolic risk.

**Central illustration:**
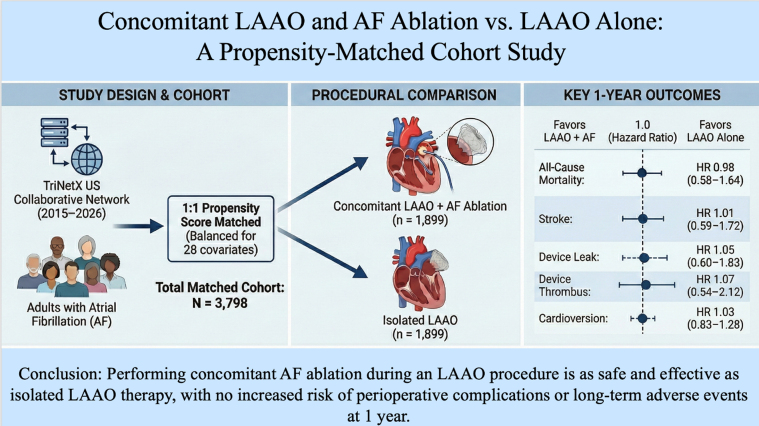
Concomitant AF Ablation and LAAO: Safety Outcomes Study design and key outcomes of concomitant atrial fibrillation (AF) ablation plus left atrial appendage occlusion (LAAO) vs LAAO alone. Using the TriNetX U.S. Collaborative Network, patients with AF undergoing LAAO were identified and stratified by concomitant same-day AF ablation. After 1:1 propensity score matching, balanced cohorts were compared across early (7-day), intermediate (90-day), and long-term (1-year) outcomes. The illustration summarizes the analytic framework and main findings, demonstrating no significant differences in mortality, ischemic stroke, device-related thrombus, or pericardial complications between groups. These findings support the procedural safety of a concomitant strategy in appropriately selected patients, with comparable short- and long-term outcomes relative to LAAO alone.

### Future perspectives

The favorable safety profile observed in this large, real-world analysis supports continued evolution toward integrated procedural strategies for AF management. Future investigations should focus on prospective, randomized comparisons of concomitant vs staged AF ablation and LAAO to better define optimal patient selection, procedural sequencing, and long-term clinical benefit. Technological advances are also likely to further refine the combined procedural approach. The growing adoption of pulsed field ablation, with its shorter procedure times and favorable safety profile, may make concomitant AF ablation and LAAO more efficient and reproducible while minimizing left atrial injury and edema. Improvements in LAAO device design, imaging modalities, and real-time intraprocedural assessment may further reduce the risk of device-related thrombus and peridevice leak.

### Study Limitations

Several limitations should be acknowledged. First, this was an observational study and remains susceptible to residual confounding despite rigorous propensity score matching. Second, outcomes were ascertained using diagnosis and procedure codes within an electronic health record-based database, which may introduce misclassification bias. Third, granular procedural details including ablation modality, lesion set, LAAO device type, intraprocedural imaging guidance, and postprocedural antithrombotic strategies were not available. As a result, our findings should be interpreted as pragmatic, real-world estimates across evolving practice patterns rather than technology-specific outcomes.

In addition, although our analysis identified patients undergoing AF ablation and LAAO on the same index date, the procedural sequence (ablation-first vs occlusion-first) could not be determined. This distinction may be clinically relevant, as emerging evidence from the COMBINATION trial suggests that an occlusion-first strategy is associated with improved outcomes,^16^ potentially by mitigating ablation-related ridge edema that can affect device sizing and positioning. Device-related outcomes, including peridevice leak, were identified using structured diagnosis and procedure codes, and detailed imaging data (e.g., transesophageal echocardiography or computed tomography), leak severity, and routine surveillance protocols were not available. Accordingly, subclinical or small leaks may be under-reported, and the observed device leak rates likely underestimate true incidence and should be interpreted cautiously. Finally, the database does not provide cause-specific mortality or procedural attribution, limiting the ability to determine whether deaths were related to underlying comorbidities or the index procedure.

These limitations underscore the need for prospective studies with detailed procedural data to further refine optimal combined procedural strategies.

## Conclusions

In this large, real-world, propensity-matched analysis, concomitant AF catheter ablation performed at the time of LAAO was not associated with increased periprocedural, short-term, or 1-year risks of mortality, thromboembolic events, or device- and pericardial-related complications compared with LAAO alone. When integrated with prior observational and randomized evidence, these findings support the procedural safety and feasibility of a combined approach in carefully selected patients. Ongoing and future prospective studies will be essential to further refine patient selection, procedural sequencing, and long-term clinical outcomes.

## Funding support and author disclosures

The authors have reported that they have no relationships relevant to the contents of this paper to disclose.

## References

[1] Benjamin E.J., Muntner P., Alonso A. (2019). Heart disease and stroke statistics—2019 update: a report from the American Heart Association. Circulation.

[2] Staerk L., Wang B., Preis S.R. (2018). Lifetime risk of atrial fibrillation according to optimal, borderline, or elevated levels of risk factors: cohort study based on longitudinal data from the Framingham Heart Study. BMJ.

[3] Joglar J.A., Chung M.K., Armbruster A.L. (2024). 2023 ACC/AHA/ACCP/HRS guideline for the diagnosis and management of atrial fibrillation: a report of the American College of Cardiology/American Heart Association Joint Committee on Clinical Practice Guidelines. J Am Coll Cardiol.

[4] Fang M.C., Go A.S., Chang Y. (2010). Warfarin discontinuation after starting warfarin for atrial fibrillation. Circulation.

[5] Wazni O.M., Saliba W.I., Nair D.G. (2025). Left atrial appendage closure after ablation for atrial fibrillation. N Engl J Med.

[6] Goldsweig A.M., Glikson M., Joza J. (2025). 2025 SCAI/HRS clinical practice guidelines on transcatheter left atrial appendage occlusion. J Society Cardiovasc Angiography Interv.

[7] Labori F., Bonander C., Persson J., Svensson M. (2021). Clinical follow-up of left atrial appendage occlusion in patients with atrial fibrillation ineligible of oral anticoagulation treatment—a systematic review and meta-analysis. J Interv Card Electrophysiol.

[8] Sawalha K., Alakchar M., Mamas M.A., Johnson D., Bhan A., Goldsweig A.M. (2026). Safety of cardioversion without anticoagulation in patients' status post left atrial appendage occlusion: a systematic review and meta-analysis. Cardiovasc Revasc Med.

[9] Alzahrani A., Wazni O.M., Hussein A. (2024). Outcomes of concomitant atrial fibrillation ablation and left atrial appendage closure: a retrospective single-center experience. JACC Adv.

[10] Phillips K.P., Romanov A., Artemenko S. (2020). Combining left atrial appendage closure and catheter ablation for atrial fibrillation: 2-year outcomes from a multinational registry. EP Europace.

[11] Chen M., Sun J., Wang Q.S. (2022). Long-term outcome of combined catheter ablation and left atrial appendage closure in atrial fibrillation patients. Int J Cardiol.

[12] Li F., Sun J.Y., Wu L.D., Hao J.F., Wang R.X. (2021). The long-term efficacy and safety of combining ablation and left atrial appendage closure: a systematic review and meta-analysis. J Cardiovasc Electrophysiol.

[13] Freeman J.V., Varosy P., Price M.J. (2020). The NCDR left atrial appendage occlusion registry. J AmColl Cardiol.

[14] Jiang Y., Li F., Li D. (2020). Efficacy and safety of catheter ablation combined with left atrial appendage occlusion for nonvalvular atrial fibrillation: a systematic review and meta-analysis. Pacing Clin Electrophysiol.

[15] Saliba W., Nair D., Swarup V. (2025). Comparison of left atrial appendage closure and oral anti-coagulation after catheter ablation for atrial fibrillation: Concomitant and sequential cohorts of the OPTION randomized controlled trial. Heart Rhythm.

[16] Du X., Chu H., Yang B. (2024). Strategy optimization for a combined procedure in patients with atrial fibrillation: the COMBINATION randomized clinical trial. JAMA Network Open.

